# Mixed signals: The effect of conflicting reward- and goal-driven biases on selective attention

**DOI:** 10.3758/s13414-017-1322-9

**Published:** 2017-04-24

**Authors:** Daniel Preciado, Jaap Munneke, Jan Theeuwes

**Affiliations:** 1 Vrije Universiteit Amsterdam, Amsterdam, Netherlands; 2 Aysel Sabuncu Brain Research Center, Bilkent University, Ankara, Turkey; 3 Department of Psychology, Bilkent University, Ankara, Turkey

**Keywords:** Attentional bias, Reward, Goal, Conflict, Integration, Selective attention

## Abstract

Attentional selection depends on the interaction between exogenous (stimulus-driven), endogenous (goal-driven), and selection history (experience-driven) factors. While endogenous and exogenous biases have been widely investigated, less is known about their interplay with value-driven attention. The present study investigated the interaction between reward-history and goal-driven biases on perceptual sensitivity (*d*’) and response time (RT) in a modified cueing paradigm presenting two coloured cues, followed by sinusoidal gratings. Participants responded to the orientation of one of these gratings. In Experiment 1, one cue signalled reward availability but was otherwise task irrelevant. In Experiment 2, the same cue signalled reward, and indicated the target’s most likely location at the opposite side of the display. This design introduced a conflict between reward-driven biases attracting attention and goal-driven biases directing it away. Attentional effects were examined comparing trials in which cue and target appeared at the same versus opposite locations. Two interstimulus interval (ISI) levels were used to probe the time course of attentional effects. Experiment 1 showed performance benefits at the location of the reward-signalling cue and costs at the opposite for both ISIs, indicating value-driven capture. Experiment 2 showed performance benefits only for the long ISI when the target was at the opposite to the reward-associated cue. At the short ISI, only performance costs were observed. These results reveal the time course of these biases, indicating that reward-driven effects influence attention early but can be overcome later by goal-driven control. This suggests that reward-driven biases are integrated as attentional priorities, just as exogenous and endogenous factors.

Selective attention is often described as the ability to effectively allocate limited information processing resources to examine specific stimuli in the continuous stream of sensory input, at the expense of ignoring other, less relevant stimuli. Traditionally, it is assumed that attentional selection is determined by two kinds of biases (Posner, 1980; Posner & Cohen, 1984): An *exogenous* bias (stimulus driven), which automatically orients attention towards physically salient events, and an *endogenous* bias (goal driven), in which attention is volitionally directed towards stimuli relevant for the present goals and motivations of the observer (for reviews, see Carrasco, 2011; Petersen & Posner, 2012; Theeuwes, 2010). In addition to these classic attentional biases, a third bias category has recently been proposed, known as *selection history*, describing how acquired knowledge and previous experiences with a certain stimulus influence the way this stimulus is perceived and interacted with (Awh, Belopolsky, & Theeuwes, 2012; Le Pelley, Mitchell, & Beesley, 2016; Munneke, Hoppenbrouwers, & Theeuwes, 2015).

The influence of value-driven biases on attention, in the context of selection history, has been demonstrated in studies using conditioning techniques to associate a neutral, innocuous stimulus with a rewarding or aversive outcome. These studies have demonstrated that stimuli signalling the possibility of gratifying or threatening events have an important influence on attentional control, with effects comparable to those exhibited by both exogenous and endogenous biases: On one hand, value-associated stimuli induce rapid, reflexive orienting responses similar to those elicited by physically salient stimuli (exogenous capture; Anderson, Laurent, & Yantis, 2011b; Failing & Theeuwes, 2014; MacLean & Giesbrecht, 2015b; Schmidt, Belopolsky, & Theeuwes, 2014). On the other hand, value-driven attentional biases have been observed to persist for extended periods of time, to be resistant to habituation and extinction, and even to transfer across different tasks (Anderson & Yantis, 2013; MacLean & Giesbrecht, 2015a; Stankevich & Geng, 2015). Moreover, value-driven biases are capable of enhancing or interfering with the effects of exogenous and endogenous attention. Specifically, stimuli associated with a certain value, rewarding or threatening, have been shown to facilitate detection and attentional selection in tasks where they are presented as attentional targets or task-relevant stimuli. Conversely, they are known to interfere and hinder voluntary attentional selection when they are presented as task-irrelevant distractors (Anderson et al., 2011b, 2013; MacLean & Giesbrecht, 2015a; Munneke, Belopolsky, & Theeuwes, 2016; Preciado, Munneke, & Theeuwes, 2016).

For instance, studies using physically salient stimuli associated with high monetary rewards (compared to equally salient stimuli associated with low or no reward) report that high-value stimuli attract attention in an automatic, involuntary fashion, leading to faster response times when acting as targets but to slower ones when used as distractors in a search display. Crucially, these effects cannot be explained in terms of saliency alone, as similar results are not elicited by equally salient stimuli not associated with reward. These findings indicate that the value associated with a stimulus enhances its perceived saliency (Anderson, Laurent, & Yantis, 2011a; Failing & Theeuwes, 2014; MacLean & Giesbrecht, 2015b; Theeuwes & Belopolsky, 2012). Importantly, these value-driven attentional effects are observed to act against endogenous attentional control determined by the goals of the observer, the specific demands of the ongoing task, or even despite the fact that in certain experimental conditions these stimuli are no longer predictive of reward (Anderson et al., 2011a; Hickey, Chelazzi, & Theeuwes, 2010; MacLean, Diaz, & Giesbrecht, 2016; Munneke et al., 2016; Munneke et al., 2015).

A recent study investigating the effect of reward-associated distractors on a visual search task (Feldmann-Wüstefeld, Brandhofer, & Schubö, 2016) corroborated that task-irrelevant distractors associated with reward can impair target processing, indicating that the extent of this interference is strongly affected by the physical salience of the distractor relative to the perceptual context it is presented in. In this visual search study, reward-associated stimuli were presented as distractors while participants had to identify and report the orientation of a tilted line embedded in a larger search array. Importantly, the search array could be homogeneous (e.g., all vertical lines), thus making both target and distractor stimuli more salient, or heterogeneous (lines in various orientations), thus making targets and distractors less salient. Findings from this study indicate that reward-associated distractors are more likely to capture attention when they are embedded in heterogeneous displays. This finding suggests that the interference of distractors, and the extent to which they can be suppressed, depends not only on their value association or the goals and motivations of the observer but also on the perceptual load or complexity of the scene; demonstrating the interplay between stimulus-, goal- and value-driven biases.

These observations suggest that the prioritization and selection of attentional targets depends on the integration of different bias categories, including the physical salience of a stimulus, the goals and intentions of an observer, and any prior experience with the stimulus, such as its value-association (Awh et al., 2012; Brosch, Pourtois, Sander, & Vuilleumier, 2011; MacLean & Giesbrecht, 2015a; Vuilleumier, 2014). Bias integration as the defining principle determining attentional selection has already been proposed in the competitive integration model (Godijn & Theeuwes, 2002), a framework describing how the programming of eye movements results from the integration of exogenous and endogenous biases within a single, common spatial priority map used to define attentional targets. According to this model, the ongoing selection and subsequent processing of attentional targets and distractors depends on the combination of the physical properties of the stimuli in the display and the specific volitional goals of the observer.

Importantly, studies testing the competitive integration model have revealed important time-course differences in the integration of exogenous and endogenous biases, showing that stimulus-driven effects are processed and integrated faster than endogenous ones (Godijn & Theeuwes, 2002; Meeter, Van der Stigchel, & Theeuwes, 2010; Trappenberg, Dorris, Munoz, & Klein, 2001). Interestingly, similar findings have been documented in studies using reward-associated stimuli, suggesting that value-driven attentional biases take effect rapidly and automatically, like exogenous biases, while goal-driven attentional control appears to require additional time to relate the available sensory input with any existing strategic goals in order to elicit an appropriate response (MacLean & Giesbrecht, 2015a; Mulckhuyse & Theeuwes, 2010; Theeuwes & Belopolsky, 2012). For example, in a recent visual search study by Failing, Nissens, Pearson, Le Pelley and Theeuwes (2015), it was shown that the fastest saccades went to a colour distractor that signalled the possibility of receiving a reward while slower saccades typically went to the coloured target. It was concluded that reward biases visual selection at the early stage of processing in an exogenous automatic way against the intentions of the observers (see also Nissens, Failing, & Theeuwes, 2016).

Considering the similarities between the attentional effects of value-driven, exogenous, and endogenous biases, the present study was set up to examine the extent to which value-driven attentional biases (specifically, reward) are integrated on the same common attentional priority map where exogenous and endogenous influences converge to guide attentional selection. From this perspective, in the current study, attentional effects were estimated by examining how reward- and goal-driven biases influence perceptual sensitivity (*d*’) and response time (RT). Experiment 1 was designed to establish how cues that signal the availability of reward would affect attentional selection, when there was no explicit voluntary goal other than to respond to the target. The aim was to corroborate whether a value-associated informative cue influences the allocation of attentional resources, resulting in performance benefits whenever a target stimulus appears at the same spatial location as the cue signalling reward and costs whenever they appear at the other location. Experiment 2 used the exact same design, except that in this version the cue that signalled reward availability also indicated that the target was most likely to appear at the location opposite from where the cue was presented. In this way, reward- and goal-driven biases are in competition, making it possible to determine if and how voluntary control can overcome reward-associated biases. Both of these experiments are based on a modified version of the Posner spatial cueing paradigm (Posner, 1980) designed to investigate performance costs and benefits in attentional orienting. A similar design was used in a recent study aimed to investigate the effects of threat-associated stimuli on attentional control (Preciado et al., 2016), with one critical difference: In the mentioned study, the stimulus–threat association is introduced by means of a classical fear conditioning procedure in an independent phase, previous to executing the cueing task. Consequently, participants had no control over the delivery of the shock, and the design is comparable to other studies where reinforcer associations are established in a training phase, and then evaluated in a testing phase where the stimulus-reinforcer contingency is removed (Anderson et al., 2011a, 2011b; Anderson & Yantis, 2013). Consequently, with this design it is plausible that the observed attentional effects are confounded by extinction effects. In contrast, in the present study, the reward-association was explicitly indicated at the beginning of the task, and then further reinforced on a trial-by-trial basis via visual feedback.

Previous studies investigating how multiple attentional biases are combined in guiding attention typically use experimental designs where different signals are independently associated with single attentional biases, and then when presented simultaneously evaluate the extent to which one bias interferes with the other, resulting in a competition at both perceptual and attentional levels (Engelmann, Damaraju, Padmala, & Pessoa, 2009; Feldmann-Wüstefeld et al., 2016; Le Pelley, Pearson, Griffiths, & Beesley, 2015; Munneke et al., 2015; Stankevich & Geng, 2014). For example, Anderson et al. (2011b) showed that stimuli associated with reward during training keep on interfering during testing even when these stimuli are no longer rewarding, demonstrating that reward has a powerful effect on attention, independent of physical saliency or endogenous goals.

These previous studies have examined the competition between stimuli that are independently associated with different attentional biases, how this competition is resolved, and how the biases are ultimately aggregated. While this approach is suited to estimate the influence of reward-associated (but otherwise task-irrelevant) stimuli on the search for a target stimulus, it is less adequate to investigate how the attentional system deals with conflicting biases which are not perceptual in nature. Indeed, in the current study, a single informative cue was associated with either a single reward-driven bias (Experiment 1) or with discordant reward- and goal-driven biases (Experiment 2). This design permits the verification of value-driven attentional capture by reward signals (Experiment 1) and the evaluation of the effects of an attentional conflict in the absence of any physical, perceptual competition (Experiment 2), in the sense that the single stimulus is expected to trigger both attentional biases. As such, there are no physical factors intervening in resolving the attentional conflict. This feature of our design stands in contrast to earlier studies where different stimuli are associated with different task properties (e.g. simultaneously presented task-relevant targets and task-irrelevant distractors). The set-up of Experiment 2 provides a unique opportunity to investigate how the opposing biases which are both task relevant and associated with the same cue affect attentional selection. It allows examining how the attentional system is able to resolve conflicting goal- and reward-driven biases, in the absence of any form of perceptual differences.

## Experiment 1

This experiment aimed to corroborate that a reward signalling cue can influence the allocation of attentional resources. If stimuli that signal reward capture attention, it is expected that when a target is presented at the location of the cue there are performance benefits (faster RT or higher *d*’) relative to a neutral condition where reward is not available. Similarly, if the cue and the target are presented at opposite locations, we expected performance costs relative to a neutral condition. Finding both costs and benefits indicates that attention was oriented to the location that contained the stimulus that signalled reward. Alternative explanations such as interference by the reward signalling stimulus not due to shifts of attention (also known as filtering costs; see Kahneman, Treisman, & Burkell, 1983) are highly unlikely when both performance costs and benefits are found (Failing & Theeuwes, 2014).

### Method

#### Ethical statement

The experimental methods and procedures for both experiments were reviewed and approved by the Scientific and Ethical review committee of the Faculty of Behavioral and Movement Sciences of the Vrije Universiteit Amsterdam, an in line with the declaration of Helsinki. Participants signed a written informed consent form before taking part in the study.

#### Participants

Students from the Vrije Universiteit Amsterdam volunteered to participate in exchange for a monetary reimbursement. Depending on their performance during the task, participants could obtain a bonus reward of maximum €5, in addition to the standard compensation for participation (€8). All participants reported having normal or corrected-to-normal vision, without colour vision impairments or any psychiatric, psychological or neurological condition. Thirty-one participants participated in Experiment 1 (14 female, 17 male; age 24.72 ± 3.51 years).

#### Stimuli and materials

Each participant was tested in a dimly lit cubicle, seated 75 cm away from a 21-in.' computer screen with the head positioned on a chin rest. Responses were collected through a standard keyboard. The experiments were designed and conducted using MATLAB (Version R2014a) and the Psychophysics Toolbox (Brainard, 1997; Kleiner, Brainard, & Pelli, 2007; Pelli, 1997). Stimuli used for the cue and target displays in both experiments consisted of circles with a diameter of 2.5° of visual angle presented bilaterally flanking the central fixation point at 6.5° of eccentricity. For the cue display, stimuli were solid colour discs (isoluminant red, green or blue; 30 cd/m2), while for the target display they were sinusoidal gratings (5 cycles/degree spatial frequency, Gaussian envelope, contrast 50%), presented either vertically (nontarget) or tilted 10° to the left or the right (target). Counterbalanced across participants, one of the used colours served as an informative cue signalling the availability of reward in this experiment, while the remaining two colours were used as neutral, uninformative cues. All colour combinations were presented equally often, such that the informative cue was presented in two thirds of the trials. Furthermore, all cueing conditions were randomly intermixed within blocks.

On each trial, the presence of the informative cue signalled the possibility of receiving a reward on that particular trial (i.e., 10 points) if a fast, correct response was given. Crucially, the reward-associated cue did not provide any spatial information regarding the location of the subsequent target, which was equally likely to appear on either location, rendering it irrelevant for the successful completion of the task. In other words, there was no incentive to voluntarily attend to the location of the reward-associated cue. These conditions were made explicit to the participants at the beginning of the experiment verbally and via written instructions, and reinforced throughout the experiment via trial-by-trial feedback indicating the accuracy of the response and whether reward was obtained or not. To reinforce the association between the informative cue and the availability of reward, points were granted for correct responses on 80% of the trials featuring the informative cue. Rewards were not delivered on every trial in order to maximize the effect of reinforcement, in line with the fundamental principles of instrumental conditioning. Specifically, it has been noted that continuous reinforcement may result in faster learning, but also in faster extinction or habituation. Conversely, intermittent variable reinforcement schedules with a high reinforcement rate result in an equally strong conditioning that is more resistant to these effects (Sander & Scherer, 2009).

#### Design and procedure

**Fig. 1:**
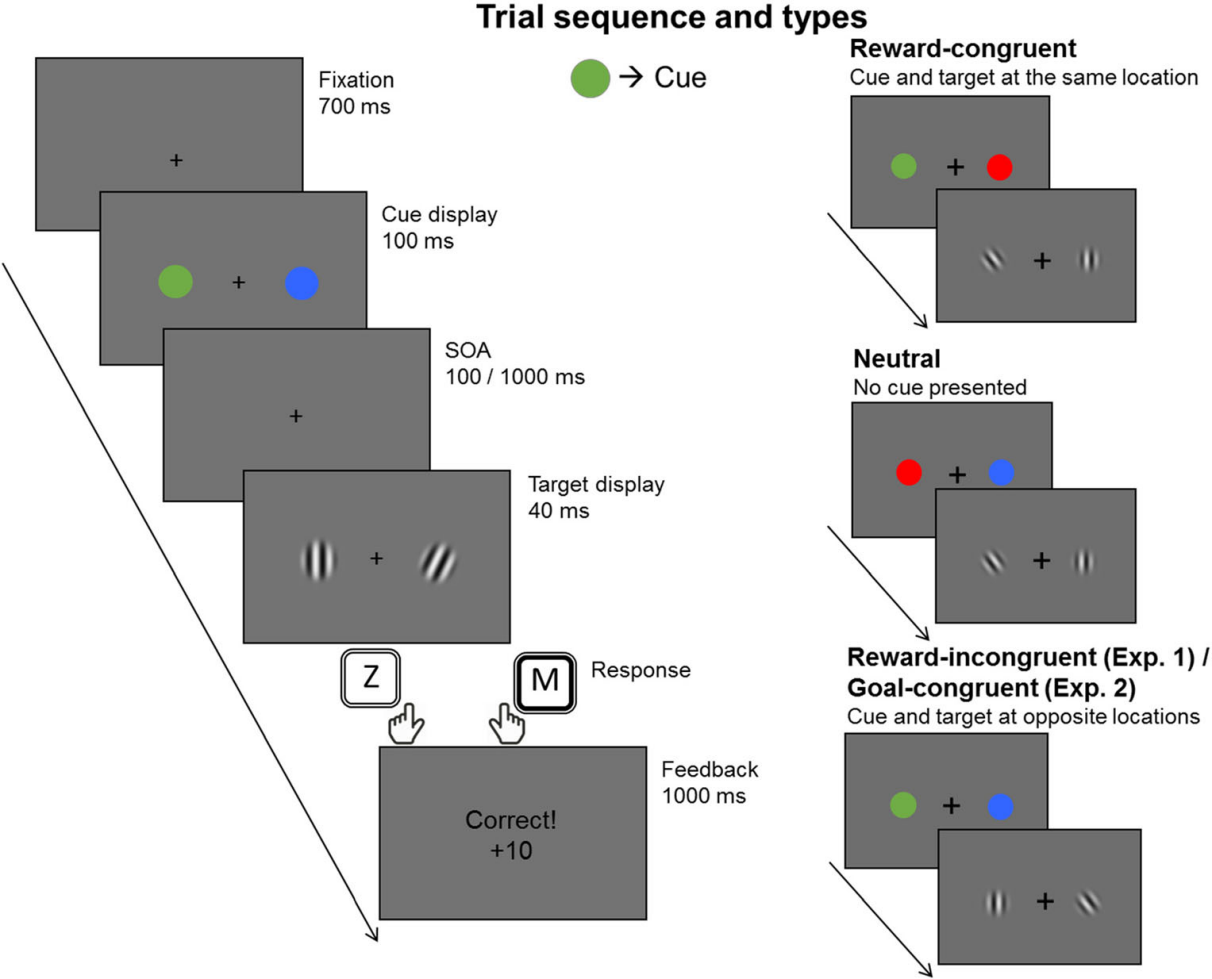
Experimental design. Left: Sequence of events in a single trial. Right: Different trial types used in both tasks. The colour of the cue was counterbalanced across participants. Note that the condition where cue and target appear at opposite locations has been labelled differently for Experiment 1 (reward incongruent) and Experiment 2 (goal congruent), in concordance with the main experimental manipulations introduced on each experiment

Starting with a central fixation cross displayed for 700 ms, a cue display containing two coloured circles was presented for 100 ms, followed by a blank screen presented for a variable interstimulus interval (ISI) duration (100 ms or 1,000 ms), introduced to examine the time course of value-driven attentional effects. After this delay, the target display containing a tilted (target) and a vertical (nontarget) grating was presented for 40 ms. Participants were instructed to indicate the orientation of the target as fast and accurately as possible by pressing *Z* if it was tilted towards the left or *M* if it was tilted towards the right. After each response, a feedback display was presented for 1,000 ms, indicating whether the response was correct, incorrect, or too slow. A schematic overview of a typical display sequence on a trial is presented in Fig. 1.

Responses were considered correct only if the target’s orientation was accurately reported within 600 ms after target display onset. Response times longer than that resulted in a *too slow* feedback, and were considered incorrect. Additionally, on trials containing the reward-signalling cue, the feedback display also indicated whether any points were earned (“+10” if a correct response was given, or “Missed +10” if response was incorrect. Note that participants could not lose points, only fail to obtain them). The next trial followed after an interval of 500 ms after the offset of the feedback display.

Participants were instructed to keep their gaze focused on the central fixation cross throughout the experiment. A break was given after each block (six blocks in total, 96 trials per block), and participants were instructed to fully take advantage of them to minimize exhaustion. Each break had a minimum duration of 10 seconds, after which the participants indicated via key press whenever they were ready to continue. In addition to the trial-by-trial feedback, participants were able to see their accumulated accuracy and the amount of points earned up to that moment during each break. Each participant completed a total of 520 trials, of which the first block (40 trials) was meant as practice and thus not considered in the analysis. Of the remaining 480 trials, each ISI condition (100 ms or 1,000 ms) was presented equally often. Depending on the location of the cue and the target, trials were classified as *reward congruent* if both cue and target appeared at the same location, *reward incongruent* if they appeared opposite from one another, and *neutral* if the informative cue was not presented (see Fig. 1).

#### Statistical analysis

Data analysis is based on a repeated-measure 2 × 3 ANOVA design conducted on *d*’ and RT as dependent variables with ISI (100 ms and 1,000 ms) and congruence (reward-congruent, reward-incongruent [Exp. 1]/goal-congruent [Exp. 2], and neutral) as predictors. Regarding repeated measures ANOVA assumptions, dependent variables in both experiments were found to be normally distributed, as evaluated via the Shapiro–Wilk test. Furthermore, for cases where the sphericity assumption is violated, the *p* value of the results includes the Greenhouse–Geisser correction, presented without corrected degrees of freedom for clarity. Further exploration of the effects of ISI and congruence are conducted via paired, two-sided *t* tests, including the false-discovery rate (FDR) correction for multiple comparisons. Additionally, Cohen’s *d* was obtained for these tests as effect size estimate, calculated as the *t* statistic divided by the square-root of the sample size (Lakens, 2013). Data processing and statistical analyses were conducted on R Version 3.2.2 (R Core Team, 2016).

For all analyses, mean RT was computed for each subject and experimental condition. Similarly, a *d*’ score was calculated for each subject and experimental condition as the difference of *z*-transformed hit and false alarm rates divided by the square root of two, applying a correction factor (+0.5) to avoid hit or false alarm rates of zero (Hautus, van Hout, & Lee, 2009; Macmillan & Creelman, 2004).

### Results

Data from four participants with poor performance (below 55% accuracy) compared to the rest of the sample were excluded from the analysis, resulting in the data of 27 participants being included in the analyses (mean accuracy 77%). Practice trials were removed before analyses, as well as trials without a response (0.3% of trials). Mean RTs were calculated excluding incorrect trials (19.1%, including wrong responses and trials with an RT > 600 ms) and trials with RT faster than 2.5 standard deviations from the mean by subject and experimental condition (<1%), resulting in a 19.72% data loss. Likewise, for the calculation of *d*’, trials faster than 2.5 standard deviations (0.45%) or slower than 600 ms were removed (6.8%), resulting in a 7.57% data loss. Mean and standard deviation for each experimental condition are presented in Table 1.

**Table 1:** Descriptive statistics for Experiment 1 by interstimulus interval (100 ms; 1,000 ms) and congruence (reward congruent, reward incongruent, neutral) conditions. *SD* = standard deviation

mean ± *SD*	*n*	Reward congruent		Reward incongruent		Neutral	
		100 ms	1,000 ms	100 ms	1,000 ms	100 ms	1,000 ms
*d*’	27	1.725 ± 0.44	1.735 ± 0.62	1.562 ± 0.52	1.561 ± 0.55	1.678 ± 0.5	1.671 ± 0.53
RT	27	448.84 ± 24.4	456.7 ± 31.9	454.20 ± 28.2	459.78 ± 35.7	455.35 ± 25.8	462.1 ± 32.9

#### *d*’ results

**Fig. 2:**
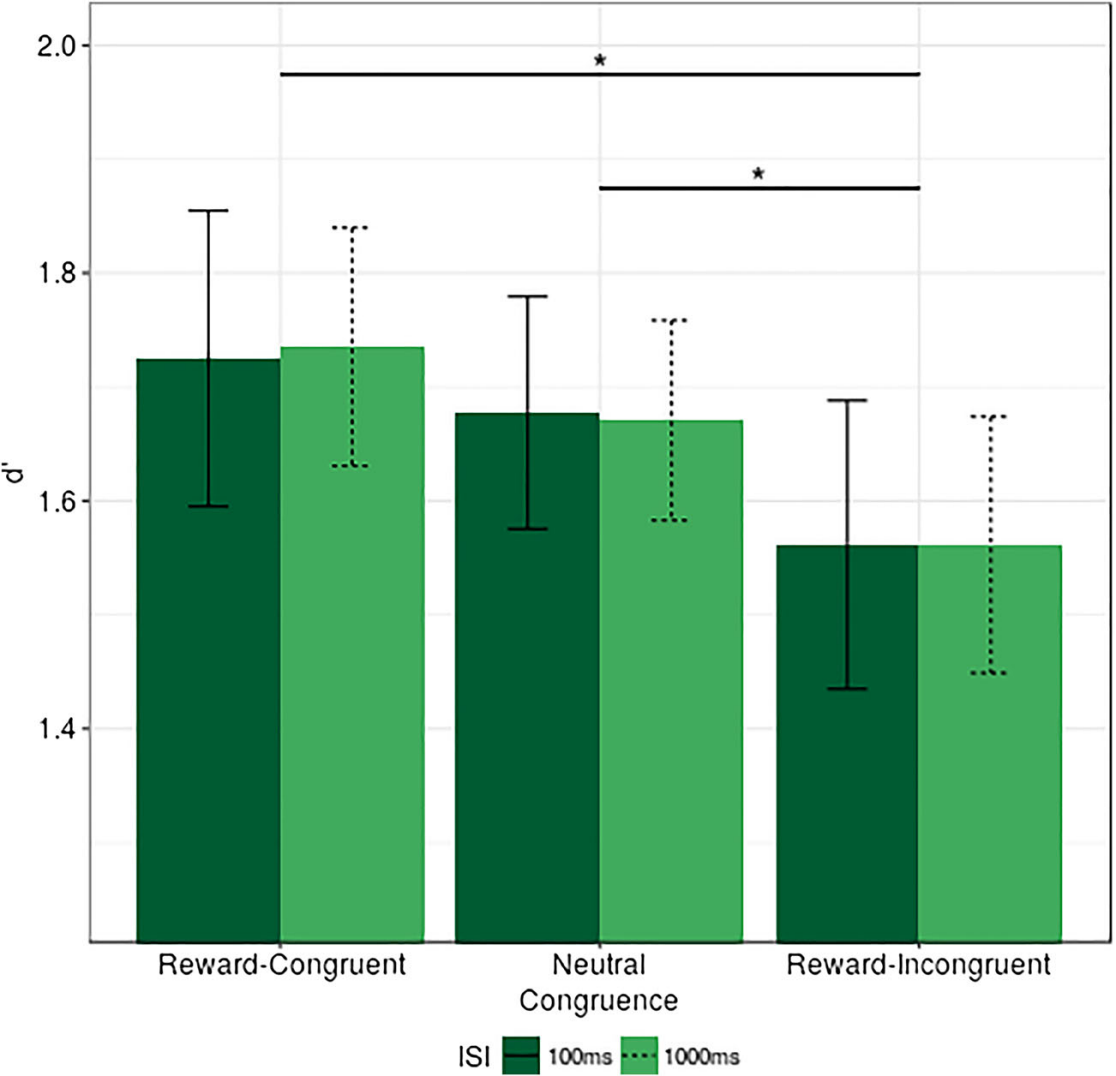
Experiment 1, *d*' by ISI and congruence, *error bars* represent within-subjects confidence intervals (Morey, 2008). *p ≤ .05. Note that the *lines* depicting significant effects represent planned comparisons (*t* tests) to investigate congruence as a main effect

The repeated-measures ANOVA conducted on *d*’ scores resulted in a significant main effect of congruence, *F*(2, 52) = 5.843, *p* = .005, η_p_^2^ = 0.02, but no effect of ISI, *F*(1, 26) = 0.0005, *p* = .98, η_p_^2^ < 0.001, nor an interaction between ISI and congruence, *F*(2, 52) = 0.012, *p* = .99, η_p_^2^ < 0.001. The further investigation of these effects via *t* tests revealed a lower *d*’ score at the reward-incongruent location, compared to the neutral reference (Δ *d*’ reward incongruent - neutral = 0.11), *t*(26) = 2.56, *p* = 0.025, Cohen's *d* = 0.45, indicative of an attentional cost on *d*’ at the reward-incongruent location. Similarly, the comparison of reward-congruent and reward-incongruent conditions was also found to be significant (Δ *d*’ reward congruent - reward incongruent = 0.17), *t*(26) = 3.065, *p* = .015, Cohen's *d* = 0.52. However, there was no reliable difference in *d*’ between reward congruent - neutral cueing conditions (Δ *d*’ reward congruent - neutral = 0.06), *t*(26) = 1.098, *p* = 0.28, Cohen's *d* = 0.21. Results are presented in Fig. 2.

#### RT results

**Fig. 3:**
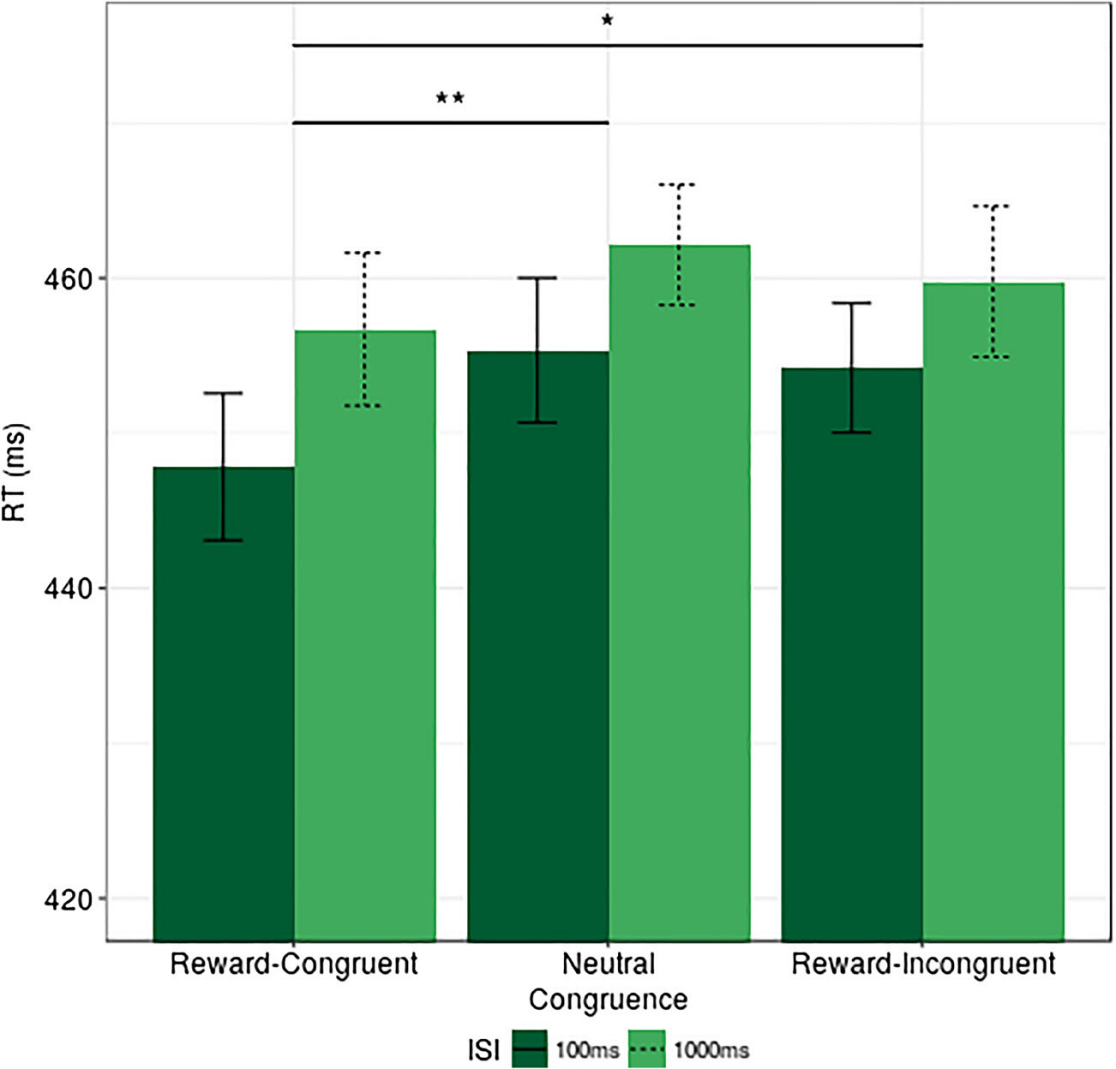
Experiment 1, RT by ISI and congruence, *error bars* represent within-subjects confidence intervals (Morey, 2008). *p ≤ .05. **p ≤ .01. Note that the *lines* depicting significant effects represent planned comparisons (*t* tests) to investigate congruence as a main effect

The analysis of mean RT resulted in significant main effects of ISI, *F*(1, 26) = 5.682, *p* = .025, η_p_^2^ = 0.014, and congruence, *F*(2, 52) = 6.734, *p* = .003, η_p_^2^ = 0.009, but no interaction, *F*(2, 52) = 0.59, *p* = .56, η_p_^2^ < 0.001. The main effect of ISI indicated that performance was significantly faster at the 100-ms ISI delay, compared to the 1,000 ms (Δ RT 100 ms - 1,000 ms = 7 ms). The analysis of congruence effects revealed that RT was significantly faster at the reward-congruent condition compared to neutral (Δ RT reward congruent - neutral = 6.3), *t*(26) = 3.468, *p* = .006, Cohen’s *d* = 0.56, an effect consistent with the notion of attentional benefits at the reward-congruent location. Nevertheless, the reward incongruent - neutral contrast did not reveal any significant differences in this condition (Δ RT reward incongruent - neutral = 1.6), *t*(26) = 0.989, *p* = .33, Cohen's *d* = 0.19. Meanwhile, a *t* test comparing reward-congruent and reward-incongruent trials returned significant results (Δ RT reward congruent - reward incongruent = 4.6), *t*(26) = 2.289, *p* = .05, Cohen's *d* = 0.41. These results are presented in Fig. 3.

### Discussion

Experiment 1 shows that attention is biased towards a stimulus that merely signals the availability of reward. Relative to the neutral condition, there were performance costs and benefits, suggesting that attention was oriented towards the location that contained the stimulus that signalled reward. Crucially, however, performance costs and benefits were not only seen at the short ISI but also at the long one, indicating that attention lingered at the location of the reward-associated stimulus, after being initially captured by it. Note that, considering the design of the experiment, there was no reason for participants to keep their attention focused at that location, as the target was equally likely to appear at either side. The initial fast capture of attention by the reward signalling stimulus is reminiscent of exogenous attentional capture, as often is seen with for example abrupt onset cues (Schreij, Theeuwes, & Olivers, 2010; Theeuwes & Godijn, 2002). Yet, unlike orienting involving exogenous cues, there is no subsequent inhibition of return (Klein, 2000), but, instead, attentional benefits remain visible at the location where attention was initially captured.

The current findings are consistent with previous studies that have used stimuli that signal threat (Preciado et al., 2016; Schmidt et al., 2016). For example, using a very similar paradigm, Preciado et al. (2016) demonstrated performance costs and benefits (in *d*’ and RT) driven by stimuli that signal threat. Crucially, in this study attention also remained focused at the location of the threat signal even after 1,000 ms, suggesting that attention dwelled at this location. Similar findings using threatening stimuli were reported by Schmidt et al. (2016) which also showed lingering attentional effects at the location of the threat. The current study provides evidence that this lingering of attention occurs not only with stimuli signalling threat but also with stimuli signalling reward. In this respect, orienting towards reward associated stimuli is very much unlike exogenous orienting towards abrupt onset cues or towards salient singletons (Theeuwes & Chen, 2005).

## Experiment 2

In Experiment 2, we wanted to determine whether attention would remain at the location signalling reward (as we saw in Experiment 1) even when there was an explicit indication to shift attention away from this location. To that end, the reward-signalling cue was indicative of the target location, signalling that it would most likely appear at the opposite side of the display. In other words, the reward-associated cue that captured attention in Experiment 1 additionally instructed participants to shift their attention away from the reward cue and to direct it towards the opposite side of the display. In line with these manipulations, Congruence conditions for Experiment 2 are defined as *reward congruent* for trials where the reward-associated cue and the target appear at the same location in the display, and *goal congruent* for trials where the target and the reward-associated cue appear at opposite locations, in concordance with the instructions provided to participants.

If the capture and dwelling of attention at the reward signalling stimulus is automatic and cannot be counteracted by voluntary control, we expect similar results as in Experiment 1. However, if voluntary control is dominant, we predict that attention is directed towards the location opposite to the reward signal. Specifically, if endogenous, voluntary control is stronger than reward-driven capture, we expect that what we label here as goal-congruent trials (where the target appears the location opposite of the reward signalling cue) show performance benefits over reward-congruent ones (where the target and the reward signalling stimulus appear at the same location). Additionally, we expect that these effects depend of the time course between cue and target, reflecting the interplay between automatic and voluntary attentional processes, and their differences in time-course (Hickey, Van Zoest, & Theeuwes, 2010; Kovach, Sutterer, Rushia, Teriakidis, & Jenison, 2014).

### Method

#### Participants

For Experiment 2, involving both reward- and goal-driven biases, 45 participants were tested (36 female, nine male; age 23.82 ± 4 years).

#### Design and procedure

Experiment 2 followed the same design and main procedures described for Experiment 1. Crucially, in this experiment the informative cue signalled two key features: First, the presence of the cue signalled the availability of a reward on that particular trial, regardless of its position. Second, the cue reliably predicted that the target was most likely going to appear at the opposite location of the display. Since both biases are associated with the same stimulus, the attentional system is presented with a priority conflict arising whenever this particular stimulus is detected. Participants were informed about these contingencies and the specific colour of the informative cue verbally and in written form at the beginning of the session. All experimental variables were manipulated independently and counterbalanced across participants.

Rewards were granted for correct responses on 80% of the trials where the cue was present, irrespective of its validity as predictor of the location of the upcoming target. Moreover, for the cue to be a reliable indicator of the target location, the target was presented on the opposite side of the display on 75% of the trials where the cue was present.

### Results

Data from three participants with poor performance (below 50% accuracy) compared to the rest of the sample were excluded, resulting in the data of 42 participants being included in the analyses (mean accuracy 77%). Practice trials were removed before analyses, as well as trials without a response (0.3% of trials). Mean RTs were calculated excluding incorrect trials (21.4%, including wrong responses and trials with an RT > 600 ms) and trials with RTs faster than 2.5 standard deviations from the mean by subject and experimental condition (<1%), resulting in 21.8% of the data being excluded from the analyses. Likewise, for the calculation of *d*’, trials faster than 2.5 standard deviations (0.4%) or slower than 600 ms were removed (7%), resulting in a 7. 7% data loss. Mean and standard deviation of each experimental condition are presented in Table 2.

**Table 2:** Descriptive statistics for Experiment 1 by interstimulus interval (100 ms; 1,000 ms) and congruence (reward congruent, goal congruent, neutral) conditions. *SD* = standard deviation

mean ± *SD*	*n*	Reward congruent		Goal congruent		Neutral	
		100 ms	1,000 ms	100 ms	1,000 ms	100 ms	1,000 ms
*d*’	42	1.39 ± 0.56	1.23 ± 0.59	1.51 ± 0.44	1.62 ± 0.47	1.63 ± 0.52	1.44 ± 0.46
RT	42	456.3 ± 28.5	458.7 ± 30.8	457.9 ± 26.9	448.6 ± 28.5	458.5 ± 25.8	458.3 ± 26.1

#### *d*’ results

The analysis of *d*’ results resulted in a marginally significant main effect of ISI, *F*(1, 41) = 4.019, *p* = .052, η_p_^2^ = 0.007, showing higher *d*’ scores at the 100ms ISI. Furthermore, a significant main effect of Congruence, *F*(2, 82) = 9.378, *p* = .007, η_p_^2^ = 0.05, and crucially, a significant interaction between ISI and congruence, *F*(2, 82) = 6.539, *p* = .002, η_p_^2^ = 0.02, were observed.

Further analysis of the interaction between ISI and congruence revealed that for the 100-ms ISI, *d*’ score was significantly lower at the reward-congruent condition, relative to the neutral (Δ *d*’ reward congruent - neutral = 0.24), *t*(41) = 3.09, *p* = .011, Cohen’s *d* = 0.43, while there were no differences between goal-congruent and neutral conditions (Δ *d*’ goal congruent - neutral = 0.12), *t*(41) = 1.79, *p* = .12, Cohen’s *d* = 0.27. These findings suggest that, at the 100 ms ISI, there were no performance benefits for a target appearing at the location of the reward signal. Moreover, a *t* test comparing reward-congruent and goal-congruent trials did not reveal reliable differences between these conditions (Δ *d*’ reward congruent - goal congruent = 0.12), *t*(41) = 1.59, *p* = .12, Cohen’s *d* = 0.24.

**Fig. 4:**
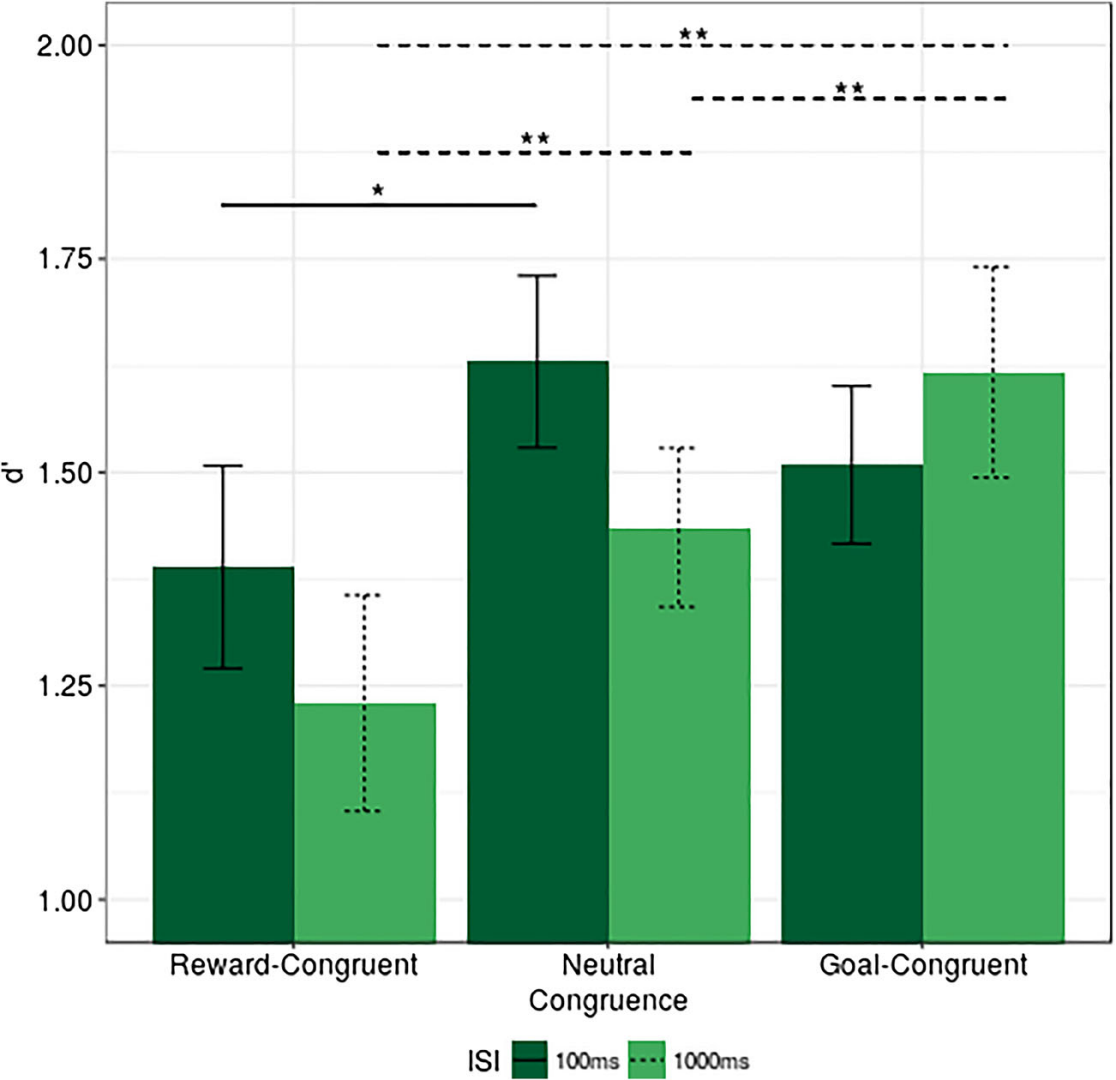
Experiment 2, *d*' by ISI and congruence, *error bars* represent within-subjects confidence intervals (Morey, 2008). *p ≤ .05. **p ≤ .01. Note that the *lines* depicting significant effects represent planned comparisons (*t* tests) to investigate the interaction between ISI and congruence. The *solid line* indicates significant contrasts at the 100 ms ISI; *dashed lines* indicate significant contrasts at the 1,000-ms condition

In contrast, at the 1,000-ms ISI condition, there were significant effects for the reward congruent - neutral comparison (Δ *d*’ reward congruent - neutral = 0.21), *t*(41) = 2.71, *p* = .01, Cohen’s *d* = 0.39, and goal congruent - neutral (Δ *d*’ goal congruent - neutral = 0.17), *t*(41) = 2.75, *p* = .01, Cohen’s *d* = 0.39, revealing that, specifically for the 1,000-ms ISI, *d*’ scores were consistently higher on goal-congruent trials but lower on reward-congruent ones. Similarly, a *t* test comparing *d*’ on reward-congruent and goal-congruent trials also turned out to be significant (Δ *d*’ reward congruent - goal congruent = 0.39), *t*(41) = 3.76, *p* = .002, Cohen’s *d* = 0.51. Results are presented in Fig. 4.

#### RT results

For RT, results revealed a significant main effect of congruence, *F*(2, 82) = 3.492, *p* = .04, η_p_^2^ = 0.007, indicating that RT was the fastest at the goal-congruent condition; and a significant interaction between ISI and congruence, *F*(2, 82) = 4.937, *p* = .01, η_p_^2^ = 0.008, but no main effect of ISI, *F*(1, 41) = 0.64, *p* = .423, η_p_^2^ = 0.002.

Further exploration of this interaction revealed that, for the 1,000-ms condition, there was a significantly faster RT on the goal-congruent trials in contrast to neutral trials (Δ RT goal congruent - neutral = 9.7), *t*(41) = 3.211, *p* = .008, Cohen’s *d* = 0.45, reflecting a goal-driven RT benefit specific to the long ISI delay. Similarly, the reward congruent - goal congruent comparison revealed reliable differences between them (Δ RT reward congruent - goal congruent = 10.13) *t*(41) = 2.22, *p* = .048, Cohen’s *d* = 0.33. However, the comparison between reward-congruent and neutral conditions did not reach significance (Δ RT reward congruent - neutral = 0.43), *t*(41) = 0.16; *p* = .88, Cohen’s *d* = 0.02.

**Fig. 5:**
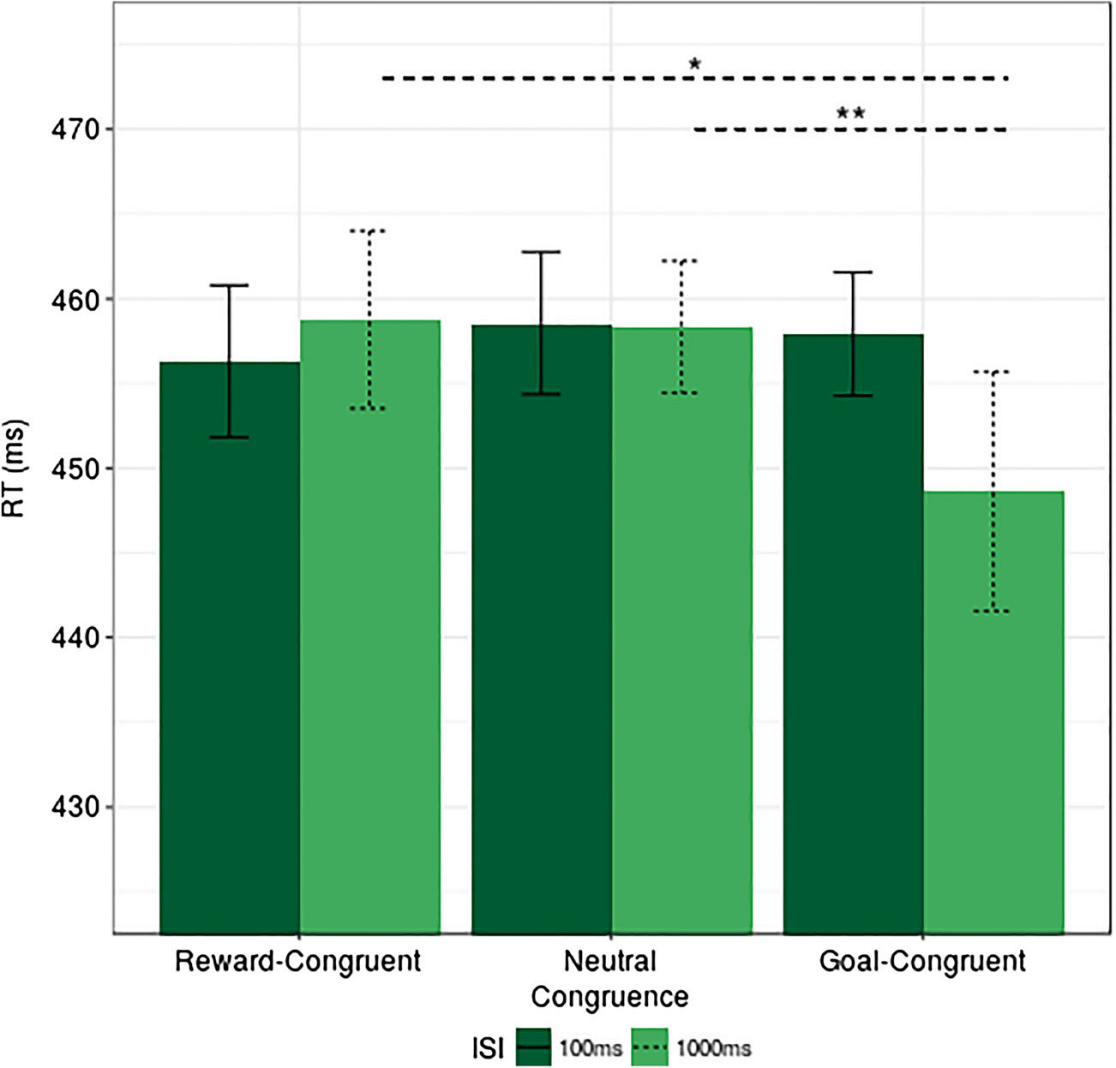
Experiment 2, RT by ISI and congruence, *error bars* represent within-subjects confidence intervals (Morey, 2008). *p ≤ .05. **p ≤ .01. Note that the *lines* depicting significant effects represent planned comparisons (*t* tests) to investigate the interaction between ISI and congruence. *Dashed lines* indicate significant contrasts at the 1,000-ms condition

Conversely, findings on the 100-ms ISI condition did not reveal any reliable differences between cueing conditions in any of the planned comparisons (Δ RT reward congruent - neutral = 2.24, *p* = .33; Δ RT goal congruent - neutral = 0.64, *p* = .72; Δ RT reward congruent - goal congruent = 1.61, *p* = 0.39). These results are summarized in Fig. 5.

### Discussion

Experiment 2 indicates that automatic reward-driven biases can be counteracted by voluntary, endogenous attentional control consistent with the task instructions. Importantly, in contrast to Experiment 1, in the current experiment there was a strong incentive to deliberately direct attention away from the reward-associated stimulus and towards the opposite location, which contained the target on the majority of trials. As reward was only granted for fast and accurate responses to the target, participants were motivated to re-direct attention away from the reward-associated stimulus as fast as possible.

At the long ISI, the observed pattern of performance costs and benefits indicates that attention was directed towards the location that is most likely to contain the target, consistent with the goal-driven attentional bias, resulting in attentional costs and benefits on *d*' and RT comparable to the classic Posner (1980) cueing effect. Crucially, even though the presentation of a reward-driven signal was expected to pull attention automatically towards its location (as in Experiment 1), the attentional costs and benefits observed in Experiment 2 at the 1,000-ms ISI indicate that this automatic tendency can be overcome by voluntary control, as attention was effectively redirected to the most likely target location, provided that enough processing time is allowed between the presentation of the cue and the target.

Interestingly, results at the 100-ms ISI show that *d*' is lower at the reward-congruent location relative to goal congruent and neutral, suggesting that the attentional priority of this location has been diminished. These findings can be explained in terms of active suppression of this location, as the cue not only signalled the reward availability but also signalled that the target was unlikely to be presented at that location. Consequently, there was every reason for observers to suppress attention towards the location of the reward signal, as it was highly unlikely to contain the target. The finding that this reduced *d*' is seen already at the 100-ms ISI condition is consistent with an automatic inhibition process, comparable in time course and automaticity to the reward-driven attentional effects. Distractor suppression as an attentional mechanism has been described in studies using cueing and visual search paradigms to investigate the extent to which reward-associated distractors interfered with task performance (Feldmann-Wüstefeld et al., 2016; Munneke, Van der Stigchel, & Theeuwes, 2008). Results from these studies indicate that the interfering effects of reward-associated distractors can be reduced if their location can be predicted (e.g., by a cue, as in Munneke et al., 2008) or if the distractor is highly salient in the environment and is unlike the search target (Feldmann-Wüstefeld et al., 2016). This demonstrates not only that the interfering effect of reward-associated cues can be reduced, but also how the attentional system integrates different sources of attentional bias in order to prioritize or devaluate potential attentional targets.

Findings from Experiment 2 could also be attributed to passive withdrawal of attention rather than active suppression, consistent with the instruction that observers should shift attention away from the reward-associated location. However, if this were the case, we would expect performance on reward-congruent trials to be similar to that observed in neutral trials. In contrast to this notion, at a ISI 100-ms condition we find a reduced *d*' on reward-congruent trials, a decrease in performance that does not concur with an improvement of *d*’ at the opposite location, a benefit that we saw only in the ISI 1,000-ms condition. Similarly, the pattern of results observed in the ISI 100-ms condition, and specifically the observation that there was no difference in *d*’ between reward- and goal-congruent conditions, could also indicate that the presence of the conflict-inducing cue results in a more general attentional impairment at early processing stages. In this sense, the results observed at the ISI 100-ms condition would represent a stage where the attentional conflict has not yet been resolved, and thus attention has not been preferentially directed towards any particular location. Nevertheless, such an account would imply that performance in the neutral condition (where no conflict is elicited) should be consistently better than the reward- and goal-congruent conditions, a result that we did not observe as goal-congruent and neutral conditions did not differ at the ISI 100 ms. Considering these alternatives, the results of Experiment 2 point out crucial differences in the integration of reward- and goal-driven attentional biases, where the observed pattern of results suggests that the attentional benefits associated with the goal-driven biases follow early effects in terms of active suppression or general attentional impairment due to an unresolved attentional conflict associated with the presence of a reward signal.

## General discussion

The present study was designed to investigate the effects of reward- and goal-driven attentional biases on *d*’ and RT in a cueing task investigating selective spatial attention with a target discrimination task. Experiment 1 examined whether a reward signalling cue would capture attention, even when it was not predictive of the target location, as the upcoming target was equally likely to appear at the same location as the cue, or at the opposite location. In Experiment 2, the informative cue not only signalled the availability of reward on that trial but also reliably predicted the most likely location of the target at the opposite side of the display.

Experiment 1 revealed performance costs and benefits congruent with a reward-driven bias, indicating that attention was oriented towards the cue that signalled the availability of reward on that trial. Moreover, the pattern of results was identical for the short and the long ISI, indicating that attention was immediately captured by the cue and then remained focused at that location even though there were no explicit reasons to do so, as the target was equally likely to appear at either location. Using a similar design, Experiment 2 showed evidence indicating a form of suppression at the short ISI, followed by a cost-benefit pattern of results favouring the goal-congruent location at the long ISI. These findings suggest that an explicit, voluntary top-down instruction (knowledge that the target would appear on the opposite side of the cue) can counteract the automatic capture of attention by the reward signalling cue.

In the current study, the results of *d*’ roughly mimics those obtained with RT. Even though previously reported effects of reward on RT (e.g., Failing & Theeuwes, 2014; Munneke et al., 2015) may represent influences of reward on relatively late decision making processes (such as response facilitation or voluntary biases), the current study demonstrating effects on *d*’ and RT already at the 100-ms ISI condition provides strong evidence demonstrating that reward associated cues are capable of modulating early perceptual processes that encode input from the visual field (Theeuwes & Van der Burg, 2007).

The results of Experiment 1 are remarkably similar to a study based on the same paradigm, but instead of signalling reward availability, the informative cue was associated with the chance of receiving an aversive electric shock, thus becoming a signal of threat, rather than reward (Preciado et al., 2016). The findings presented here indicate that reward signals, very much like threat signals, do capture attention immediately, resulting in performance costs and benefits, in favouring *d*’ and RT, favouring the cued location and already evident at the short ISI. While it is known that threatening cues capture and hold attention over a relatively longer time intervals (Fox, Russo, Bowles, & Dutton, 2001; Schmidt et al., 2016), the current study shows that this is similarly the case for cues that signal the reward availability, as demonstrated by the consistent pattern of costs and benefits observed at both the short and long ISI for Experiment 1.

While Experiment 1 showed that a cue that reward signals result in an *increased* perceptual sensitivity at the reward-congruent location (*d*’ = 1.72) relative to neutral (*d*’ = 1.67) already at the 100-ms ISI condition; in Experiment 2, that very same cue, which now also signalled that the target was unlikely to be presented at the reward-congruent location, resulted in *reduced* perceptual sensitivity at that location (*d*’ = 1.43) relative to neutral (*d*’ = 1.63). Within 100-ms ISI, the automatic capture of attention by the reward cue results in an enhanced perceptual sensitivity at the reward-congruent location in Experiment 1, yet the same cue that signals reward availability resulted in reduced perceptual sensitivity in Experiment 2, when there was a strong incentive to direct attention away from the informative cue. This finding is consistent with studies that have combined reward- and goal-driven biases (Buschschulte et al., 2014; Della Libera & Chelazzi, 2009; MacLean & Giesbrecht, 2015a). Specifically, in conditions in which reward-associated stimuli are used as distractors, observers are capable of deliberately implementing task-driven strategies to minimize the performance costs associated with them. Furthermore, the extent to which attention is oriented away from the reward-associated distractor is related to the magnitude of the reward, with higher rewards resulting in more effective control preventing the selection of the reward-associated distractor (Buschschulte et al., 2014). This evidence suggests that the deployment of attention (in terms of the selection or rejection of attentional targets) appears to be dynamically defined based not only by attentional priorities and biases, but also by behavioural relevance, in terms of the relative value of a particular behaviour (Della Libera & Chelazzi, 2009).

The notion that there is active, voluntary suppression at the short ISI Experiment 2 is consistent with the idea that active suppression of a location (rather than a feature) can be very efficient (see Theeuwes, 2013, for a review). This notion is related to other location-based suppression mechanism such as inhibition of return (see Klein, 2000, for a review). Yet in the current study we cannot determine whether the reduced *d*’ at the short ISI is the result of initial capture of attention followed by suppression (as in inhibition of return- IOR, for example), or whether observers actively and voluntarily inhibited a particular location by instructing them to direct attention away from that location. Given that there is already suppression evident at 100-ms ISI, it is likely that observers are able to actively suppress the location of the informative cue at early stages of visual processing. This fast suppression is consistent with recent studies that claimed that suppression of salient singletons in the additional singleton task can be feature-based (Gaspelin, Leonard, & Luck, 2015; Moher & Egeth, 2012; Sawaki & Luck, 2010; Vatterott & Vecera, 2012). Such fast suppression can also take place at the level of whole perceptual dimensions (Feldmann-Wüstefeld, Uengoer, & Schubö, 2015). Alternatively, the results from Experiment 2 could reflect the unresolved conflict between reward- and goal-driven attentional biases, possibly resulting in an impaired performance at the ISI 100-ms condition for both reward- and goal-congruent trials. While both accounts support the notion of critical differences between early and late attentional effects driven by conflicting reward- and goal-driven biases, further research is required to conclusively elucidate whether these differences, and particularly the attentional effects observed at the short ISI, are driven by early, active suppression mechanisms, or by a more general attentional impairment resulting from resolving the attentional conflict.

The current findings are consistent with the selection history bias (Awh et al., 2012), according to which attentional resources can be dynamically allocated depending on the outcome of previous encounters with a particular stimulus. In Experiment 1, in which the target was equally likely to appear at either location, attention was biased towards the reward signalling cue. In Experiment 2, the reward bias no longer attracted attention, but instead attention was driven away from the reward signalling cue towards the location that was most likely to contain the target. Note that participants were motivated to respond fast and accurately to the target, as this was required to obtain the reward that was signalled by the cue.

Together, these findings support the notion that attentional guidance and selection depends on the integration of different types of biases. This integration process is not limited to exogenous and endogenous biases, but also includes those driven by selection history. The present study demonstrates that attentional priority maps are plastic, readily updated and adapted to accommodate strategic goals and the behavioural relevance of present and previous attentional choices in order to optimize present behaviour and its consequences.
